# Targeted Therapy for Adrenocortical Carcinoma: A Genomic-Based Search for Available and Emerging Options

**DOI:** 10.3390/cancers14112721

**Published:** 2022-05-31

**Authors:** Daniel Alexander Hescheler, Milan Janis Michael Hartmann, Burkhard Riemann, Maximilian Michel, Christiane Josephine Bruns, Hakan Alakus, Costanza Chiapponi

**Affiliations:** 1 Department of Nuclear Medicine, University Hospital Münster, Albert-Schweitzer-Campus 1, 48149 Münster, Germany; daniel.hescheler@ukmuenster.de (D.A.H.); burkhard.riemann@ukmuenster.de (B.R.); 2 European Institute for Molecular Imaging (EIMI), University of Münster, 48149 Münster, Germany; 3 Department of General, Visceral, Tumor and Transplant Surgery, University Hospital Cologne, Kerpener Strasse 62, 50937 Cologne, Germany; milan.hartmann@smail.uni-koeln.de (M.J.M.H.); christiane.bruns@uk-koeln.de (C.J.B.); costanza.chiapponi@uk-koeln.de (C.C.); 4 Institute of Zoology, University of Cologne Germany, Zuelpicher Str. 47 B, 50674 Cologne, Germany; mmichel8@uni-koeln.de

**Keywords:** targeted molecular therapy, new treatment advances, Human Genome Project, adrenocortical carcinoma

## Abstract

**Simple Summary:**

Adrenocortical carcinoma is a rare disease for which in silico analysis may help to identify therapy options. Here, we look at the potential of already FDA-approved drugs and therapies in clinical trials based on genes mutated in ACC and genomic alterations known to be targeted by these drugs. Overall, 67% of the ACCs presented in silico drugability, especially for new emerging options such as TP53-modulating drugs or Wnt signaling pathway inhibitors. These results might help plan future clinical trials.

**Abstract:**

In rare diseases such as adrenocortical carcinoma (ACC), in silico analysis can help select promising therapy options. We screened all drugs approved by the FDA and those in current clinical studies to identify drugs that target genomic alterations, also known to be present in patients with ACC. We identified FDA-approved drugs in the My Cancer Genome and National Cancer Institute databases and identified genetic alterations that could predict drug response. In total, 155 FDA-approved drugs and 905 drugs in clinical trials were identified and linked to 375 genes of 89 TCGA patients. The most frequent potentially targetable genetic alterations included TP53 (20%), BRD9 (13%), TERT (13%), CTNNB1 (13%), CDK4 (7%), FLT4 (7%), and MDM2 (7%). We identified TP53-modulating drugs to be possibly effective in 20–26% of patients, followed by the Wnt signaling pathway inhibitors (15%), Telomelysin and INO5401 (13%), FHD-609 (13%), etc. According to our data, 67% of ACC patients exhibited genomic alterations that might be targeted by FDA-approved drugs or drugs being tested in current clinical trials. Although there are not many current therapy options directly targeting reported ACC alterations, this study identifies emerging options that could be tested in clinical trials.

## 1. Introduction

Adrenocortical carcinoma (ACC) is a rare but very aggressive disease. The vast majority of patients display either locally invasive disease (stage III) or metastatic disease (stage IV) at diagnosis [1]. Even after R0 resection, 50–80% of patients reportedly develop recurrent or metastatic disease [2]. The EDP-M schedule (etoposide, doxorubicin, and cisplatin plus oral mitotane), which is currently the first-line chemotherapy for locally advanced or metastatic ACC offers a median progression-free survival (PFS) of 5.1 months [3]. Further, half of the patients receiving first-line EDP-M will require additional treatments in the 6 months following treatment failure [4]. There are no established targeted treatment options in ACC, and consequently, clinical trials represent the only options for these patients.

Recent studies performed whole-exome sequencing (WES) on 45 and 91 ACC samples [5,6]. The authors report a mean somatic mutation rate of 0.6 and 0.9 mutations per megabase. This shows that ACC is a tumor with a very low mutation burden, compared with other human cancers with mutation rates as high as 45.2 mutations per megabase (skin squamous cell carcinoma) [7].

The molecular characterization of ACC to date uncovered the beta-catenin pathway as a potential target for therapies, in particular TP53, APC, CTNNB1, CDKN1C, IGF-2, NF1, RB1, and menin gene alterations as possible targets [8]. Beta-catenin seems to play a major role in both localized and advanced or metastatic ACC, which additionally displays ERBB4, GPCR, RAR, and PDGFR as further possible targets [9]. However, in spite of a recent study suggesting that more than a half of 364 ACC tumors may show actionable mutations based on OncoKB [10], clinical studies testing drugs that target the vascular endothelial growth factor (VEGF) pathway [11], the epidermal growth factor receptor (EGFR) pathway [12,13], the insulin-like growth factor 1 receptor (IGF-IR) pathway [14] or the mammalian target of rapamycin (mTOR) pathway [15,16] were discouraging so far [17,18,19,20].

The present study was designed to specifically find which of the 155 currently Food and Drug Administration (FDA)-approved drugs could be adapted in ACC. Further, we investigated not-yet-approved targeted therapy options that might play a role in ACC based on the TCGA genomic data. This may provide a systematic framework for future clinical studies since ACC is sufficiently rare to render large-scale clinical studies a practical impossibility.

## 2. Materials and Methods

All data were obtained from open-access databases and referenced (cBioPortal [21,22], OnkoKB [23], My Cancer Genome [24], and CIVICS [25]).

### 2.1. Linking FDA-Approved Targeted Therapy and Targeted Therapy in Clinical Trials to Their Genetic Targets

#### 2.1.1. FDA-Approved Targeted Therapy

FDA-approved drugs for any cancer therapy were identified by searching the databases of the National Cancer Institute [26] My Cancer Genome [24] and linked to their genetic target(s), as previously described [27].

#### 2.1.2. Targeted Therapy Drugs in Current Clinical Trials

**Figure 1:**
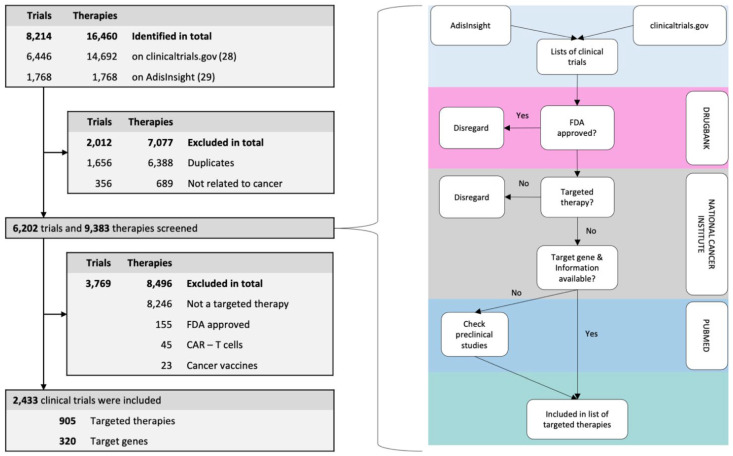
Flow diagram of methodological proceed acquiring non-FDA-approved drugs.

The flow diagram of our methodological approach is shown in Figure 1 and displays our criteria to identify targeted therapies in clinical trials:

The search engines ClinicalTrials.gov (accessed on 1 October 2021) [28] and AdisInsight [29] were screened for clinical trials that had been started after 1 January 2015. For the database ClinicalTrials.gov [28] the following search phrases were used: “target(ed)” and/or “mutation/mutated” plus one of the following: “cancer”, “tumo(u)r”, and “neoplasm” in different constellations. Trial duplicates (1656), as well as trials not related to cancer (356), were excluded. For each clinical trial, all employed drugs and each therapy were checked in the database of DrugBank [30] to check whether it was a targeted therapy and whether it was already approved by the FDA. In the next step, we checked the target gene or protein in the drug dictionary of the National Cancer Institute (NCI) [26] for the target mechanism. If all these conditions were applied to the targeted therapy, it was added to the list of targeted therapies in clinical trials. Vaccines, as well as immunotherapies such as CAR T-cell treatments, were not included. All targeted therapies were linked to targeting genes and their characteristics, consistent with the procedure used for the FDA-approved drugs.

### 2.2. Identification of Altered Druggable Genes in Adrenocortical Carcinoma (ACC)

#### 2.2.1. Alterations in Druggable Genes of ACC Patients

The TCGA data of Zheng et al. [10] were used as the source for genomic analysis. In total, 89 patients with complete datasets of identified mutations and putative copy numbers were included/Of these, 70 patients with stage I-III, 17 patients with stage IV (distant metastases) and 2 patients were not specified. Further, the genomic analysis focused on genes that were linked to drugs, as described above. Data were generated using Mutsig (Broad Institute of Massachusetts Institute of Technology and Harvard University, Cambridge, MA, USA) and GISTIC 2.0 (Broad Institute, University MIT and Harvard, Cambridge, MA, USA) and were acquired through the DataBank cBioPortal.

#### 2.2.2. Gain of Function in Druggable Oncogenes

Gene alterations resulting in a gain of function in an oncogene were identified using the databases CIVIC [25] and OncoKB [23]. Gene alterations were defined as “gain of function” as follows: OncoKB score (gain of function or like gain of function), CiViC score (pathogenetic, likely pathogenetic or positive), as well as mutations within Chang’s mutational hotspots [31].

#### 2.2.3. CNV Amplification in Druggable Oncogenes

The algorithm GISTIC 2.0 by cBioPortal [21,22] classified the copy number level per gene as follows: “−2”, deep loose; “−1”, shallow loose; “0”, diploid; “1”, low-level gain; and “2”, high-level amplification. The threshold high-level amplification “2” was chosen due to the significant increase in CNVs.

#### 2.2.4. Mutation Hotspot Analysis

The mutation datasets were screened to detect mutation hotspots, their frequency, and druggability. Mutation variants known to be responsive to FDA-approved drugs according to the database DOCM [31] were also searched.

### 2.3. Drug Response Prediction

#### 2.3.1. Gene Alterations Affecting Drug Response

Mutation variants and CNVs directly or indirectly affecting genes of potentially targeted treatments were identified by querying My Cancer Genome [24], CIVIC [25], TARGET [32] and OncoKB [23]), as previously described [27].

#### 2.3.2. Drug Response Prediction

The potential genetic drug response was predicted by integrating the genetic target of each drug with data on patient tissue samples annotated with biologically relevant genetic alterations (as described previously [33]) and was defined as follows:

(a) Gain of function in an oncogene;
(b) CNV amplification in an oncogene;
(c) Specific genetic alterations in an oncogene;
(d) Genetic alterations in tumor suppressor genes.

Whether a patient might respond to a given drug was predicted based on the following criteria:

(i) The gene underlying the drug target shows a copy number increase;
(ii) The drug targets a gene whose product shows a gain of function;
(iii) A specific alteration with known drug effectiveness is present.

Sensitivity or resistance to the targeted therapy was defined by mining My Cancer Genome [24], CiViC [25], TARGET [32], and OncoKB [23]. The following criteria were used to handle overlapping data:

Level A CiViC level A and B, FDA indication, My Cancer Genome, OncoKB level 1, 2a, R1;

Level B CiViC level C and D, OncoKB level 3a;

Level C Targeted Database all levels, CiViC level E, OncoKB level 4.

In case both a score for “sensitivity” and one for “resistance” occurred at the same level, the drug was scored as resistant. Where different levels of evidence conflicted, we decided based on the next higher level of evidence (A > B > C). If a final score of “resistance” occurred, the drug was excluded as a potential candidate [33].

### 2.4. Ethics

This study was conducted in accordance with the provisions of the Declaration of Helsinki and local laws, as previously described [27].

## 3. Results

### 3.1. Targeted Therapy and Linked Genetic Targets

In total, 155 FDA-approved drugs were included in this study. Out of 8214 trials and 16,460 therapies, we identified an additional 905 drugs that have not yet been approved. A total of 1060 drugs were linked to 375 genes (Supplementary Tables S1 and S2).

### 3.2. Genetic Alterations in ACC—The TCGA Dataset

We identified genetic alterations potentially targetable by these 1060 drugs in the TCGA dataset. These included 20% genetic alterations in TP53, 13% CNV-Amp in BRD9, 13% CNV-Amp in TERT, 13% gain-of-function mutation in CTNNB1, 7% CNV-Amp in CDK4, 7% CNV-Amp in FLT4, and 7% CNV-Amp in MDM2. Less frequently observed alterations included genes such as ATM, FGFR4, HDAC 7 and 9, NOTCH 1 and 3, and NTRK 1 (Supplementary Table S3).

**Figure 2:**
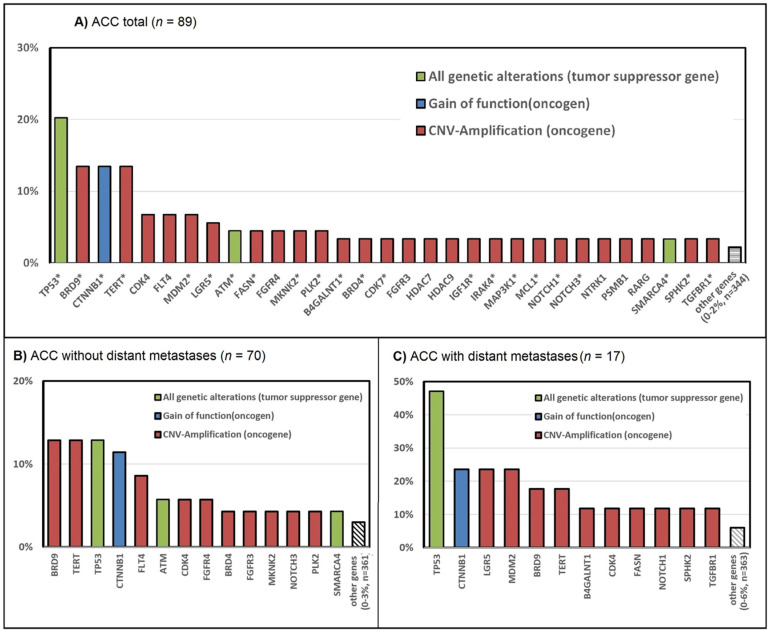
Target genes with FDA-approved drugs and drugs in clinical trials (**A**) depicts genetic alterations of TCGA (x-axis), which are targeted by FDA-approved drugs or drugs currently being tested in clinical trials (*). On the y axis, the frequency of these alterations can be read. Gain-of-function alterations are depicted in blue, and CNV amplifications, in red. Green is used for tumor suppressor genes; (**B**,**C**) depict a subgroup analysis of non-metastatic ACC (**B**) versus metastatic ACC (**C**). In 2 of 89 patients, tumor stage was not documented (*n* = 87).

In stage IV ACC tumors, there was a higher rate of TP53 mutations (47% versus 13%), and of CTNNB1 gain-of-function mutations (24% versus 11%), along with a higher rate of LGR5 and MDM2 CNV amplifications (24% versus 1%, and 14% versus 3%, respectively, compared with localized tumors. BRD 9 and TERT were altered in 18% of advanced and 13% of localized tumors (Supplementary Table S4). Figure 2 summarizes these differences.

### 3.3. Mutation Hotspots Based on the TCGA Data

High mutation rates were observed in the following hotspots: CTNNB1 S45* (eight patients), CTNNB1 G34* (three patients), KMT2A D877* (two patients), POLE T457* (two patients), TP53 E339* (two patients) (Supplementary Table S5). None of these mutation hotspots is currently druggable.

### 3.4. CNV Co-Amplification Pairs Based on the TCGA Data

All BRD9 amplifications occurred together with TERT amplifications (12/12 patients) probably due to the genomic proximity (5p15.33). Other CNV co-amplifications occurred in LGR5-MDM2 (*n* = 5), BRD4-NOTCH3 (*n* = 3), BRD9-FGFR3 (*n* = 3), CDK4-HDAC7 (*n* = 3), CDK4-MDM2 (*n* = 3), CDK7-MAP3K1 (*n* = 3), FGFR3-TERT (*n* = 3), and FGFR4-FLT4 (*n* = 3) (Supplementary Table S5).

### 3.5. Potential Genomic Drug Response

**Figure 3:**
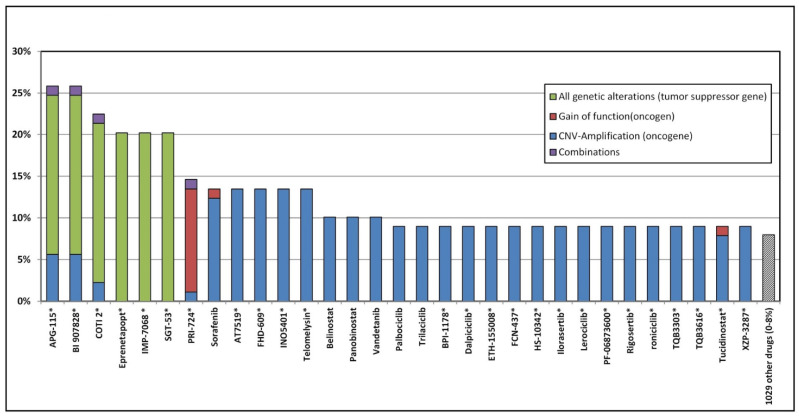
Potential drug options. This bar chart lists the potential therapeutic options of FDA-approved drugs or drugs currently being tested in clinical trials (*) in the ACC cancer cohort based on tumor genetics. APG-115, BI 907828, COTI 2, Eprenetapopt, IMP-7068, and SGT-53 are drugs acting on TP53 via different mechanisms (APG-115, BI 907828 indirectly, while COTI2 additionally targets AKT). PRI-724 targets CTNNB1, Telomelysin ION5401, and FHD-609 TERT and BRD9, which were found to be frequently co-altered. Histone deacetylase (HDAC) inhibitors, such as Belinostat, Panobinostat, or Tucidinostat, multikinase inhibitors such as Sorafenib and Vandetanib, and CDK inhibitors such as Palbociclib/Trilaciclib/Dalpiciclib are further predicted options.

The following drugs show a genetic alteration in ACC that may respond to therapy (Supplementary Table S6): MDM2 inhibitors and p53 reactivators APG-115 and BI 907828 (currently tested in clinical studies) showed a predicted response in 26% of tumors. The p53 reactivator COTI 2 might be efficacious in 22% of tumors, while the other p53 reactivator Eprenetapopt, along with the Wee1 kinase inhibitor IMP-7068 and SGT-53 (a cationic liposomes complex encapsulating human wild-type p53 DNA in a plasmid backbone) may work in 20% of ACC tumors. The CTNNB1 targeting drug PRI-724 had targets in 15% of tumors, while a response to the TERT inhibitors Telomelysin and INO5401 was predicted for 13% of tumors. The CDK inhibitor AT7519, the selective BRD9 protein degrader FHD-609 and the multikinase inhibitor Sorafenib showed to be targeted in 13% of cases. For 10% of cases, we saw a predicted response to the multikinase inhibitor Vandetanib. The histone deacetylase (HDAC) inhibitors Belinostat/Panobinostat, and the CDK inhibitors Palbociclib/Trilaciclib/Dalpiciclib showed a potential response in another 9–10% of tumors (Figure 3).

## 4. Discussion

ACC is a rare disease, with 0.7–2 cases per million individuals [34], and it is aggressive, with two-thirds of these tumors being either grade III or grade IV at diagnosis [35]. The Median PFS of patients with locally advanced or metastatic disease receiving the currently recommended first-line schedule is 5.1 months [8], and there are few options after fist line failure. Targeted therapies are being tested in several ongoing studies. However, clinical data are slow in development due to the rarity of ACC and the scarcity of ACC patients.

For this reason, we decided to create a theoretical framework for planning future clinical studies based on available genomic data and drug response prediction, as described previously for other tumors [27,36]. A systematic in silico preclinical analysis including available and emerging options might help save time and costs and optimize treatments since most of the potential targets screened do not need to be validated experimentally.

The actionable targets among all TCGA alterations identified in this study include TP53 alterations in 20% of patients, gain-of-function mutations in CTNNB1 in 13%, CNV amplifications in TERT in 13%, CNV amplifications in BRD9 in 13%, CNV amplifications in CDK4 in 7%, CNV amplifications in FLT4 in 7%, and CNV amplifications in MDM2 in 7% (Figure 2). Other alterations occurred less frequently (LGR5 (5.7%), BRD4 (4.5%), DNMT1 (4.5%), FASN (4.5%), MCL1 (4.5%), MKNK2 (4.5%), NOTCH3 (4.5%), NTRK1 (4.5%), TGFBR1(4.5%), TYK2 (4.5%)). These alterations resulted in a predicted effect for APG-115/BI 907828 (MDM2 inhibitors and p53 reactivator) in 26%, COTI 2 (mutant p53 activator and targeting AKT1, AKT2, and AKT3) in 22%, Eprenetapopt/IMP-7068/SGT-53 in 20% (mutant p53 reactivator), PRI-724 in 15% (targeting CTNNB1, CREB1, WNT1), Telomelysin/INO5401 in 13% (targeting TERT), FHD-609 (BRD9 inhibitor) in 13%, AT7519 (multi-cyclin-dependent kinase inhibitor), in 13%, Sorafenib (multi-protein-kinase inhibitor including VEGFR, PDGFR, and RAF) in 13%, Belinostat/Panobinostat (pan HDAC inhibitor) in 10%, Vandetanib (VEGFR2 inhibitor) in 10%, and Palbociclib/Trilaciclib/Dalpiciclib (CDK 4/6 inhibitor) in 9% of tumors. TP53, CTNNB1, TERT, and BRD9 alterations were present in 47%, 24% and 18% of advanced stage IV tumors.

TP53 is a critical tumor-suppressor gene that is mutated in more than half of all human cancers. Not only do mutations impair its antitumor activity, but they can also confer oncogenic properties [37]. The p53-targeted therapy approach includes both compounds capable of reactivating wild-type p53 functions and compounds able to eliminate mutant p53. Targeting the MDM2–P53 axis for restoring p53 function with APG-115 has emerged as an attractive strategy for AML [38] and with BI 907828 for liposarcoma [39]. COTI-2 is, together with Eprenetapopt (APR-246), one of the two p53 reactivators that have entered clinical trials [40]. The drawback of these therapies is the inherent toxicity of these compounds. Thus, more targeted p53 reactivators are needed to minimize toxicity and improve the therapeutic window [37]. IMP 7068 is an inhibitor of Wee1 kinase, a cell-cycle regulator that plays important role in DNA damage response (DDR) pathways. There is a strong biological rationale supporting that Wee1 is a target mediating lethality in p53-mutant tumors [41]. SGT-53 is a complex of cationic liposomes encapsulating a normal human wild-type p53 DNA sequence in a plasmid backbone. This complex has been shown to deliver the p53 cDNA efficiently and specifically to tumor cells [37]. Although the use of ncRNAs targeting TP53 is still in its early stages, there is potential in this approach [37]. Particularly the high frequency of TP53 reported in the TCGA data for metastatic tumors supports the clinical potential of targeting this pathway.

CTNNB1 was altered in 14.8% of the TCGA samples and 28% of the foundation one tumors [14]. Interestingly, it was more frequently altered in metastatic ACC, as opposed to localized stages (Figure 2), suggesting that CTNNB1 could be involved in the switch to metastasis. On the targeting drug PRI-724, there are some data for HCC [42], breast cancer [43], colorectal cancer [44], endometrial cancer [45], sarcomas [46], and neuroendocrine tumors [47]. It has not been approved by the FDA yet and, to our best knowledge, has never been tested in ACC patients. However, the role of the Wnt/β-catenin pathway has been suggested [6], and two Wnt inhibitors (LGK974 and ETC-1922159 in combination with Pembrolizumab) are being tested on selected solid malignancies that have progressed despite standard therapy or for which no effective standard therapy exists. The results of these trials are pending.

TERT alterations were identified in 13% of ACCs. A similar rate has also been reported by Gupta et al. [48]. Studies with Telomelysin (OBP-301), a gene-modified oncolytic adenovirus introducing human telomerase reverse transcriptase (hTERT) promoter in cancer cells, and INO-5401, a combination of three separate DNA plasmids targeting Wilms tumor gene-1 (WT1) antigen, prostate-specific membrane antigen (PSMA) and human telomerase reverse transcriptase (hTERT) genes, are ongoing. Telomelysin is currently being tested in combination with Pembrolizumab on esophageal cancer (NCT03921021), HNSCC (NCT04685499), HCC (NCT02293850), and melanoma (NCT03190824). INO-5401 is being tested on glioblastoma (PMC7678727). Interestingly TERT and BRD9 were co-altered in all cases, possibly due to the close locations (5p15.33) of these two genes. This co-alteration has not been described in ACC so far. It still needs to be confirmed whether FHD-609, a highly selective protein degrader of BRD9, in combination with Telomelysin INO5401, might play a role in ACC treatment.

We also identified CDK inhibitors as a potential treatment. CDK4 was also altered in 7.7% of samples and CDKN2A in 13.5% of tumors in the cohort studied by Pozdeyev [14]. In an mRNA-based analysis by Liang et al., a CDK4 overexpression was also identified in 62% of samples [49]. Both Liang et al. and Fiorentini et al. also reported an in vitro effect of Palbociclib on ACC cell lines [49,50]. Palbociclib has been approved by the FDA for breast cancer. Clinical trials with Palbociclib in ACC have neither been performed nor registered yet. Further, there is not much literature on the not-yet-approved CDK9 inhibitor AT 7519 [51,52].

The multikinase inhibitor Sorafenib, despite a predicted response in 13% of patients in the present study, has already been tested in a multicenter phase II study with 25 patients with metastatic ACC, who showed cancer progression on mitotane and at least one prior cisplatin-based chemotherapy regimen, in combination with paclitaxel. This study was prematurely interrupted because all evaluable patients (9/9) had disease progression in their first 8 weeks [53]. The great tumor burden and the multiple previous treatment lines in these patients could have been contributory, as argued by the authors [53]. They also suggested that the combination of metronomic chemotherapy and Sorafenib, both targeting angiogenesis may have induced tumor hypoxia in the cellular microenvironment leading to accelerated tumor growth. It also needs to be considered that mitotane has a strong and long-lasting inducing effect on CYP3A4 activity, which results in clinically relevant interactions with multiple drugs, including tyrosine kinase inhibitors [54]. However, Cerquetti et al. recently analyzed its effect in vitro and recommended against its use [55]. Other multikinase inhibitors such as Sunitinib did not produce significantly better results in clinical studies: Sunitinib was administered concomitantly to mitotane in a prospective open-label phase II study with 35 evaluable patients with advanced ACC, resulting in stable disease in only 14.3% of patients. The rest either had progressive disease (24/35) or died (6/35) [56]. Axitinib delivered no objective response in an open-label, phase II trial of 13 previously treated patients [15]. Under Erlotinib in combination with gemcitabine, there was only a minor response to therapy in 1 of 10 patients with metastatic/recurrent/unresectable/locally advanced ACC who had progression on mitotane and at least 2 chemotherapy regimens, including a platinum-based regimen [17]. There was no objective response in 19 patients with unresectable ACC who had progressed on prior mitotane or chemotherapy under Gefitinib [16]. There were partial responses in 2 of 15 patients receiving Lensitinib in an open-label, phase I study [57] but no difference in median OS or PFS in a double-blind placebo-controlled phase III trial of 139 patients with locally advanced or metastatic ACC, who progressed on at least one, but no more than three, prior lines of therapy, having had received mitotane in the neoadjuvant, adjuvant, or advanced disease setting [18]. Apatinib and Cabozantinib are currently being tested in NCT04318730, NCT03612232, and NCT03370718 (Table 1). There are no data on Vandetanib in ACC; however, adrenal insufficiency in patients treated with Vandetanib for other tumors has been described and could suggest a possible side effect [58].

Histone deacetylase (HDAC) inhibitors, such as Belinostat, Panobinostat, or Tucidinostat have been tested in lymphomas [59], multiple myeloma [60], breast cancer [61], and thyroid cancer [62]. Data were rather discouraging in thyroid cancer [62]. To our best knowledge, there is no clinical experience in ACC so far.

MDM2 alterations were identified in 6.8% of the TCGA tumors and 5.8% of cases in Pozdeyev et al. [14]. A role in atopic dermatitis has been described for the MDM2-targeting Janus kinase and syk inhibitor Gusacitinib [63]. However, there is no available evidence on oncologic disease, to our best knowledge. Milademetan Tosylate (NCT03634228) and RO5045337 also have OnkoKB evidence Level 3A (“Compelling clinical evidence supports the biomarker as being predictive of response to a drug in this indication”).

NOTCH inhibitors have received much attention as therapeutics for several solid tumors [64]. NOTCH family genes are likely to play a role in immune cell modulation, mediating epithelial–mesenchymal transformation (EMT), angiogenesis, focal adhesion, and PI3K–Akt signaling [65]. The modest clinical success of current Notch-targeting strategies is mostly due to their limited efficacy and severe on-target toxicity in Notch-controlled healthy tissues [66]. Different components of the Notch1 signaling pathway are overexpressed in ACCs, which suggests a role for the pathway in malignant transformation [67]. It is still unclear if notch inhibitors will contribute to ACC in the future, possibly in combination with immunotherapy.

It needs to be mentioned that immunotherapy, although it is not the subject of the present analysis, is currently in focus for the treatment of ACC, with several studies testing checkpoint inhibitors (Table 1). Although in an international, multicenter phase Ib trial (NCT01772004), avelumab administered to 50 ACC patients previously treated with mitotane or platinum-based chemotherapy showed only a modest clinical activity (a partial response in 6% of patients, stable disease in 42%, and disease progression in 46% of patients [68]) some prolonged OS is observed in several studies [69] suggesting a benefit in a subset of patients. Besides endogenous steroid hypersecretion in functioning ACCs, glucocorticoids are also frequently prescribed as supplementation to treat adrenal deficiency in ACC patients treated with mitotane or following adrenal surgery and might interact with immunotherapies [70]. Combining the administration of immune checkpoint inhibitors with drugs targeting the Wnt-β catenin and TP53 pathways could be an attractive treatment paradigm for future studies [71]. Nivolumab and pembrolizumab are currently being tested in several studies (Table 1).

Table 1 lists the clinical trials in adrenocortical cancer, including study design, primary outcomes, and reported adverse events (adapted from Kiesewetter et al. [72] and updated with current clinical trials). Many trials do not focus on ACC exclusively but rather include ACC among other rare cancer types.

It needs to be mentioned that this study is exclusively in silico, and our results require in vitro and in vivo confirmation. Secondly, all results are based on the TCGA data, which includes 91 ACC tumors. Recently, Pozdeyev et al. presented foundation medicine data including 364 tumors [14]. However, the authors used next-generation sequencing data from foundation one, in contrast to Zheng et al., who presented whole-genome sequencing for the TCGA dataset [10]. Pozdeyev et al. further suggest CDKN2A (13.5%), NF1 (10.2%), ATM (8%), PDGFRA (4.7), PTCH1 (4.1%), BRCA2 (3.8%), KRAS (2.7%), EGFR (2.7%), NTRK2 (2.5%), TSC 1 and 2 (2.2%, respectively), and ALK (2.2%) as further targets. This discrepancy with our results could be because the authors used OnkoKB only for predicting response and considered drug effects based on pathways adding up the targets. In their analysis, they included all levels 1–4, with and without R1-2 resistances. In the present study, OnkoKB, CiViC, and My Cancer Genome were considered, and the predicted drug effects took into account that a tumor might develop a resistance to drug treatment. A further caveat is that it remains unclear if, for example, OnkoKB target level 1 agents such as the MEK inhibitor Selumetinib, which has proved very successful in neurofibromatosis, might also help the 10% of ACC patients bearing NF1 alterations: to date, Selumetinib produced no significant result in NSCLC [73,74], uveal melanoma [75] and other cancers [76], despite NF1 mutations.

Both Lippert et al. [77] and Pozdeyev et al. [14] have reported at least one druggable molecular alteration in 60% of ACCs. In contrast, when the authors reduced their data to FDA-approved drugs, Darabi et al. found only 16% of ACC patients were predicted to be responsive [78]. Further compounding this, a preliminary series of 10 ACC tumors analyzed with a broad spectrum of next-generation sequencing at the University of Wien resulted in no relevant targets identified [72]. It also needs to be mentioned that alterations found in the TCGA or any other dataset may not be causal to disease etiology or even progression, and more research needs to be carried out [79]. In line with Pozdeyev et al. [14], we found that 67% of the TCGA tumors had at least one actionable mutation.

## 5. Conclusions

In conclusion, this is a network-based analysis designed to identify possible therapeutic targets among FDA-approved and not-yet-approved currently available drugs. The aim was to optimize the planning of clinical studies by turning to data mining and investigating genomic targets of drug response and resistance and correlating this with the ACC sequencing datasets. This study identified both approved options such as Palbociclib and Trilaciclib, and new options such as TP53-targeting drugs, PRI-724, or the combination FHD-609 with Telomelysin or INO5401 as options. In vitro and in vivo data are required for verifying these results.

## Figures and Tables

**Table 1:** Studies on ACC adapted from Kiesewetter et al. [72].

Drug/ ClinicalTrials.gov ID/Reference	Mechanism of Action	Setting	Primary Outcome	Study Design	Status
Cabozantinib NCT03612232	VEGFR 1/2/3, KIT, NTRK2, FLT-3, AXL, RET, MET, and TEK.	Relapsed/refractory advanced or metastatic ACC (mitotane discontinued, serum concentration < 2 mg/L)	PFS at 4 months	Single-arm Phase II	Recruiting
Cabozantinib NCT03370718		Locally advanced or metastatic ACC (mitotane stopped for 1 month, serum concentration < 2 mg/L)	PFS at 4 months	Single-arm phase II	Active, not recruiting
Camrelizumab/Apatinib NCT04318730	Camrelizumab: PD-1 receptor Apatinib: VEGF2, KIT, SRC	Second-line treatment of recurrent or metastatic adrenocortical carcinoma	ORR	Single-arm phase II	not yet recruiting
IPI-549 (Eganelisib)/Nivolumab NCT02637531	IPI-549: PIK3C Nivolumab: PD-1 receptor	ACC locally advanced or metastatic and other advanced and/or metastatic carcinoma or melanoma, excluding sarcoma	DLT, AE	Single Arm phase I/Ib	active, not recruiting
Relacorilant/pembrolizumab NCT04373265	Relacorilant: SGRM Pembrolizumab: PD-1 receptor	Locally advanced or metastatic ACC with glucocorticoid excess (mitotane level ≤ 4 mg/L)	ORR, dose-limiting toxicities	Phase Ib	recruiting
Therapeutic vaccine (EO2401)/nivolumab NCT04187404	Nivolumab: PD-1 receptor	ACC locally advanced or metastatic (also including pheochromocytoma or paraganglioma)	Safety	Phase I/II	recruiting
ONC201 NCT03034200	MAPK1	Unresectable, recurrent, locally advanced, refractory, or metastatic neuroendocrine tumors including cholangiocarcinoma and ACC (age 14 and older)	CR, PR	Single-arm phase II	active not recruiting
Nivolumab/ipilimumab NCT03333616	Nivolumab: PD-1 receptor ipilimumab: CTLA-4 binding	Locally advanced or metastatic ACC (mitotane allowed for control or endocrine symptoms) and other rare genitourinary tumors	ORR	Single arm phase II	Recruiting
Nivolumab/ipilimumab NCT02834013		Relapsed/refractory advanced or metastatic ACC or other rare tumors	ORR	Single-arm, phase II	Recruiting
Pembrolizumab NCT02721732	Pembrolizumab: PD-1 receptor	Relapsed/refractory advanced or metastatic ACC or other rare tumors	Non-progression at 27 weeks, adverse events	Single-arm phase II	active, not recruiting
Pembrolizumab/lenvatinib NCT05036434	Pembrolizumab: PD-1 receptor Lenvatinib: *VEGFR, PDGFR*, *EGFR*, *RET*, *KIT*	Advanced ACC after failure of platinum- and mitotane-based chemotherapy	ORR	single-arm phase II	Not yet recruiting

Abbreviations: AE, adverse event; ACC, adrenocortical carcinoma; AXL, AXL receptor tyrosine kinase; CR, complete response; CTLA-4, cytotoxic T-lymphocyte antigen-4; DLT, dose-limiting toxicities; *FTL-3*, FMS-like receptor tyrosine kinase-3; KIT, encoding for the tyrosine kinase c-KIT; MAPK1, mitogen-activated protein kinases 1; MET, encoding for c-Met, also called tyrosine-protein kinase Met or hepatocyte growth factor receptor HGFR; NRTK2, neurotrophic receptor tyrosine kinase 2; ORR, objective response rate; PD-1, programmed cell death protein 1; *PDGFR*, platelet-derived growth factor receptor; PR, partial response; *RET*, rearranged during transfection; SGRM, selective glucocorticoid receptor modulator; TEK, TEK receptor tyrosine kinase; *VEGFR*, vascular endothelial growth factor receptor.

## Data Availability

The datasets generated during and/or analyzed during the current study are available in the figshare repository, http://doi.org/10.6084/m9.figshare.19531561 accessed on 29 May 2022.

## Supplementary Materials

The following supporting information can be downloaded at: https://www.mdpi.com/article/10.3390/cancers14112721/s1, Table S1: List of FDA-approved drugs; Table S2: List of drugs in clinical trials; Table S3: Results of genetic alterations in target genes of targeted therapy drugs in ACC; Table S4: Results of subgroups ACC with and without distant metastases; Table S5: Potential genetic therapy options of targeted therapy drugs in ACC; Table S6: Results of CNV-Amplification gene pair and mutation hotspots in ACC.
